# Cell Retention as a Viable Strategy for PHA Production from Diluted VFAs with *Bacillus megaterium*

**DOI:** 10.3390/bioengineering9030122

**Published:** 2022-03-16

**Authors:** Milos Kacanski, Lukas Pucher, Carlota Peral, Thomas Dietrich, Markus Neureiter

**Affiliations:** 1 Department of Agrobiotechnology, Institute of Environmental Biotechnology, University of Natural Resources and Life Sciences, Vienna, Konrad-Lorenz-Str. 20, 3430 Tulln, Austria; milos.kacanski@boku.ac.at (M.K.); lukas.pucher@students.boku.ac.at (L.P.); 2 TECNALIA, Basque Research and Technology Alliance (BRTA), Parque Tecnólogico de Álava, Leonardo Da Vinci 1, 01510 Minano, Spain; carlota.peral@tecnalia.com (C.P.); thomas.dietrich@tecnalia.com (T.D.)

**Keywords:** polyhydroxyalkanoates, poly(3-hydroxybutyrate), cell retention, volatile fatty acids, *Bacillus megaterium*

## Abstract

The production of biodegradable and biocompatible materials such as polyhydroxyalkanoates (PHAs) from waste-derived volatile fatty acids (VFAs) is a promising approach towards implementing a circular bioeconomy. However, VFA solutions obtained via acidification of organic wastes are usually too diluted for direct use in standard batch or fed-batch processes. To overcome these constraints, this study introduces a cell recycle fed-batch system using *Bacillus megaterium* uyuni S29 for poly(3-hydroxybutyrate) (P3HB) production from acetic acid. The concentrations of dry cell weight (DCW), P3HB, acetate, as well as nitrogen as the limiting substrate component, were monitored during the process. The produced polymer was characterized in terms of molecular weight and thermal properties after extraction with hypochlorite. The results show that an indirect pH-stat feeding regime successfully kept the strain fed without prompting inhibition, resulting in a dry cell weight concentration of up to 19.05 g/L containing 70.21% PHA. After appropriate adaptations the presented process could contribute to an efficient and sustainable production of biopolymers.

## 1. Introduction

There is a growing crisis related to the depletion of non-renewable resources and their impact on the environment. A way to address this challenge is to utilize waste materials from existing processes to decrease the environmental impact and create new products. To achieve this, it will be necessary to develop processes for valuable products that are either already on the market or have the capacity to be environmentally friendly alternatives to existing solutions. One approach is to produce biodegradable and biocompatible materials from renewable resources. In this regard, polyhydroxyalkanoates (PHAs) are good candidates because of their material properties [1] and lower impact [2,3] on the environment.

PHAs are intracellular polymers that help bacteria and archaea survive environmental challenges [4] and they are produced when there is an excess of available carbon and a simultaneous limitation of an essential nutrient such as nitrogen or phosphorous [5]. They are elastomeric or thermoplastic polyesters that are biocompatible and biodegradable [1]. Since their constitutive monomers influence their properties, they are broadly classified as either short chain length (3–5 carbon atoms in the monomer backbone), medium-chain length (6–14 carbon atoms in monomer backbone) or long-chain length PHAs (more than 14 carbon atoms) [6,7]. Exact material properties are also determined by polymer aspects such as average molecular weight and polydispersity index [1]. The most basic and most common type of PHA is poly(3-hydroxybutyrate) (P3HB), a highly crystalline and brittle material with thermoplastic behavior [8].

Current production schemes for PHA involve raw materials that are either unsustainably expensive or they divert resources from food production [2,3,9]. It is clear that the right way forward for PHA is to utilize cheap waste materials for the production and recently this concept has received a lot of attention. Various raw materials such as grass silage [10], crude glycerol [11], surplus whey [12] as well as chicory root hydrolysate [13], and desugarized molasses [14] have been identified as suitable for production. Notably, production from volatile fatty acids (VFAs) has also been successfully demonstrated in various cases and scenarios, including pure as well as mixed culture approaches [15,16,17,18,19,20]. VFAs are very promising substrates for PHA production, since—in contrast to wastes with specific composition—they can be readily obtained from multiple wastes through already established digestion processes. Sludges from municipal wastewater are available in abundant volumes and there is rapid development for their use for VFA production [21]. In addition, food waste is also disposed in significant quantities and often studied for its VFA potential [22]. Industrial wastes, e.g., from agricultural, dairy, pulp and paper industries are also evaluated as perspective sources of VFAs [23,24]. Chemicals that can be derived from multiple renewable sources are deemed as platform chemicals within the biorefinery concept [25] and developing production processes around VFAs fits well within this context. They are already on the market to a significant extent [26], and there is also substantial research to optimize their output from renewable sources [22,26,27]. This aspect would also contribute to the robustness of PHA production, as constant availability of raw material throughout the year is less of an issue if it can be obtained from various sources.

The critical disadvantage of renewable carbon sources for PHA biosynthesis is that they are usually too diluted for productive batch and fed-batch processes [12,13,28,29,30,31]. A straightforward approach to tackle this issue would be to concentrate the substrates. However, this makes the process less favorable from an economic and ecological perspective, due to the additional energy input and costs of the concentration step and the risk of accumulating various substances that may inhibit microbial growth [9,13,30]. There is an alternative approach in the form of a cell recycle reactor, where the working volume is kept constant, with all the biomass in it, while the substrate flows through the process, according to the selected feeding regime. This has been demonstrated in a study with a recombinant *E.coli* fed by whey [28] and similar approaches employed *Cupriavidus necator* to produce PHA with carbohydrates [32,33]. Membrane-based biopolymer production with organic acids is mentioned only in one study with food scraps digestate that contained mostly lactic and butyric acid [34]. Volatile fatty acids, in particular, have an additional constraint, since they can inhibit the producing microbes [34,35]. However, this can be solved by implementing a properly controlled feeding regime. Cell recycling is a powerful approach for working with diluted carbon sources, especially when the desired product is accumulated inside the cells. Ultimately, the concentrated cell mass will result in high productivities, which could be further increased by establishing a fully continuous process.

Regarding the active culture, there is a choice between processes based on aseptic pure cultures and mixed microbial consortia (MMC). The MMC approach is generally cheaper and more robust. There is no requirement for sterility, and the adaptation of the microbial consortium is a necessary and expected part of the process. This allows for flexibility in substrate quality, which is an important aspect in fed-batch approaches from complex waste-based substrates. However, MMC processes come with certain drawbacks, which call for the development of pure culture processes. The MMC approach requires more effort to guarantee specific material properties related to the composition and distribution of monomeric units and molecular weight and related aspects such as crystallinity [20,31,36]. In addition, pure cultures offer more potential with regard to the use of metabolic and genetic engineering for improving all aspects important for PHA production.

However, there is a possibility to transfer some advantages of the MMC approach to pure culture processes with certain design choices. The impact of dissolved inhibitive substrate components [31] is less relevant in cell recycle processes, as they are less likely to accumulate due to washout. Furthermore, the use of extremophilic production strains can reduce to some extent the dependence on successful sterilization and proper protection from contamination during the long processes [9]. *Bacillus megaterium* uyuni S29, a halophilic organism isolated from a Bolivian Salt Lake, has the potential to adequately address this function. This strain was already reported to accumulate P3HB at relatively high content (up to 60% of dry cell weight) [37], including a study with waste-based media [14].

The objective of this work was to establish P3HB production from a VFA-based substrate using *Bacillus megaterium* uyuni S29 as the production strain. The process was designed as a cell recycle fed-batch system with an indirect pH-stat feeding regime.

## 2. Materials and Methods

### 2.1. Microorganisms and Media for Cultivation

The working strain in this study, *Bacillus megaterium* uyuni S29 (CECT 7922), was cultivated for preculture on nutrient broth (NB) agar plates (5 g/L peptone from meat, 3 g/L meat extract, 15 g/L agar-agar) at 30 °C. For long term storage, the organism was kept at −80 °C as cryo-stock in modified beef extract medium (10 g/L peptone from casein, 10 g/L meat extract, 5 g/L NaCl) with 10% glycerol.

The preculture was inoculated from a plate and cultivated in a rotary shaker (Infors Multitron) at 35 °C and 130 rpm for 24 h in 300 mL baffled shake flasks containing 100 mL of fermentation media. To achieve the goals of this study, we designed a synthetic medium containing acetate as the main carbon and energy source alongside some additional nutrients in the form of yeast extract and citric acid (present only at the beginning). These nutrients were added to speed up initial growth as they are known to promote growth of *Bacillus megaterium* strains [38]. Acetate content was kept below a value of 5 g/L to avoid inhibition. The medium composition was as follows: 10 g/L NaCl, 1.5 g/L KH_2_PO_4_, 4 g/L Na_2_HPO_4_, 0.025 g/L CaCl_2_, 0.025 g/L NH_4_Fe(III) citrate, 0.04 g/L MgSO_4_·7H_2_O, 1 mL/L of 5 × SL6 trace elements solution, 5 g/L (NH_4_)_2_SO_4_, 5 g/L yeast extract, 0.75 g/L citric acid and 4 g/L sodium acetate. The 5 × SL6 trace elements solution contained: 0.5 g/L ZnSO_4_·7 H_2_O, 0.15 g/L MnCl_2_·4 H_2_O, 1.5 g/L H_3_BO_3_, 1g/L CoCl_2_·6 H_2_O, 0.05 g/L CuCl_2_·2 H_2_O, 0.1 g/L NiCl_2_·6 H_2_O and 0.15 g/L Na_2_MoO_4_·2 H_2_O. pH was set to 7.0 and the media was autoclaved at 121 °C for 20 min. Phosphates and ammonium sulfate were autoclaved separately to avoid precipitation.

Consumed acetate was replenished via the feed medium (10 g/L NaCl, 0.5 g/L KH_2_PO4, 1 g/L Na_2_HPO_4_, 0.025 g/L, CaCl_2_, 0.025 g/L NH_4_Fe(III) citrate, 1 mL/L of 5 x SL6 trace elements solution and 20 g/L Na acetate, pH: 7.0). The medium was designed to induce nitrogen limitation by excluding (NH_4_)_2_SO_4_ and yeast extract. A concentration of 20 g/L sodium acetate (14.63 g/L of acetic acid) was chosen to mimic the concentration of carbon source expected from acidification processes. Phosphate content in the feed was decreased to evade precipitation. The acid control solution was 2 M H_2_SO_4_ with the addition of 8 g/L of MgSO_4_·7 H_2_O to evade precipitation. The first batch of feed media was sterile filtered in a 10 L bottle. Subsequent batches were not sterilized, since there was only a low risk for contamination due to low nitrogen and high acetate content.

### 2.2. Fermentation Setup

**Figure 1:**
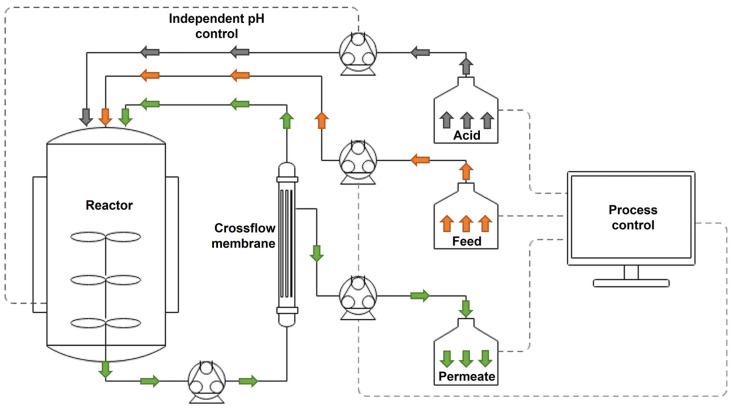
Schematic representation of the cell recycle system. Addition of acid (grey) and VFA feed (orange) and removal of permeate (green) via pumps is recorded by scales and controlled by the process control system in order to keep process values stable and maintain a constant volume in the reactor.

The experiments were conducted in a fermenter with 5 L working volume (B. Braun Biotech International GmbH, Melsungen, Germany) operating at a working volume of 4 L. Aeration (2–10 L/min) and stirring (200–800 rpm) were controlled stepwise by feedback from the dissolve oxygen (DO) probe against a setpoint of 20% DO. The reactor was connected with the membrane system, as shown in Figure 1. We used a 0.02 mm pore size microfiltration membrane (CFP-2-E-4MA, 420 cm^2^ membrane area, GE healthcare, Amersham, UK) sterilized by flushing 2 M NaOH for 2 h. Before connecting to the reactor, NaOH was removed by flushing with sterile water. During the process, a crossflow pump was operated at 1750 mL/min. Transmembrane pressure was measured before the membrane as well as on the permeate side. The pumps for the crossflow and the permeate were set to automatically reverse the flow every 20 min for 1 min to avoid intensive fouling of the membrane by the biomass.

Feed, permeate removal and pH were programmed with a control system (1769 CompactLogix Controller, Rockwell Automation, Milwaukee, WI, USA) to pump out the permeate, as measured by the weight of the permeate bottle, at the exact amount of liquid supplied via the feed and acid control, as measured by the respective weights of feed and acid solution bottles. Since the change in pH in the reactor is the result of acetate consumption, the feed was linked to the pH control by following the current addition rate of acid solution. A control interface table provided the system with information about the current rate of feed addition based on the current acid consumption. The setpoint for the feed rate was defined to correspond to a range of acid consumption.

### 2.3. Analytical Methods

Samples of the fermentation broth were taken 3 times per day and separated into triplicates of 5 mL each, from which further analysis was performed. Samples were centrifuged (Centrifuge 5810, Eppendorf, Hamburg, Germany) at 2724× *g* for 15 min and the supernatant was frozen at 20 °C for subsequent analysis. The precipitated biomass was washed and dried for 48 h at 105 °C to determine the dry cell weight (DCW) and subsequently the P3HB content.

Acetate concentration was determined by HPLC (1100 series with refractive index detector, Agilent, Santa Clara, CA, USA; ION 300 column, Transgenomic, New Haven, CT, USA; column temperature: 45 °C; eluent: 0.005 M H_2_SO_4_, flow rate: 0.325 mL/min; pressure: 46 bar).

The total amount of nitrogen available for consumption to the strain was determined as total Kjeldahl nitrogen. Approximately 1.5 mL of fermentation supernatant was dissolved in 20 mL concentrated sulfuric acid and 1 Kjeltab (Thomson & Capper, Cheshire, UK). Following digestion in a Digest Automat K-438 (Büchi, Flawil, Switzerland), the nitrogen concentration was determined by titration with an AutoKjeldahl Unit K-370 (Büchi, Flawil, Switzerland).

Biopolymer production was quantified by oxidation to crotonic acid [39] determined by HPLC using the same setup described for the determination of acetic acid. This method was considered sufficient to quantify the produced polymer, as this strain was shown to produce only P3HB under the conditions applied in the experiments [14].

### 2.4. Polymer Extraction and Characterization

The polymer was extracted via biomass digestion with sodium hypochlorite [40]. Centrifuged biomass was suspended in 0.2 M H_2_SO_4_ and left overnight at room temperature. After 24 h, the pH value of the suspension was set to 13.0 to break up the cell membrane and after another hour a 10% solution of sodium hypochlorite was added to double the volume, yielding the final hypochlorite concentration of 5%. After one hour, the suspensions were centrifuged, and the precipitate was dried at 105 °C. The resulting powder was P3HB, which was used for further characterization.

Weight average (M_W_) and number average (M_n_) molecular weight were determined using gel permeation chromatography (GPC) PL-GPC-50 (Agilent Technologies, Santa Clara, CA, USA) equipped with an IR detector. The column was a RESIPORE 3 µm (300 × 7.5 mm) heated to 25 °C and the mobile phase was chloroform (flow rate: 1 mL/min). The standard EasiCal Polystyrene pre-prepared calibration kit, PS-2 Part Number PL2010-0605 (Agilent Technologies, Santa Clara, CA, USA) was used. The polydispersity was calculated as the ratio between the weight- and number-average molecular weights. Sample injection volumes of 20 μL were used.

Melting temperature T_M_ as well as glass transition temperature T_G_ were determined via differential scanning calorimetry (DSC). Samples (3–4 mg) were weighed in aluminum pans, which were sealed and then heated in a DSC calorimeter (TA Instruments, New Castle, DE, USA) at a constant rate of 10 °C/min from 20 °C to 220 °C, cooled down to –50 °C, and heated up again to 220 °C. An empty pan was used as a reference. Dry nitrogen was used as a purge gas at 50 mL/h.

Thermal degradation was studied by TGA using a SDT 2960 Simultaneous DTA-TGA/DSC-TGA (TA Instruments, New Castle, DE, USA). The polymer sample was heated from 20 °C to 500 °C at a rate of 2 °C/min in an active nitrogen atmosphere at a flow rate of 20 mL/min.

## 3. Results and Discussion

### 3.1. Growth and Polymer Production

**Figure 2:**
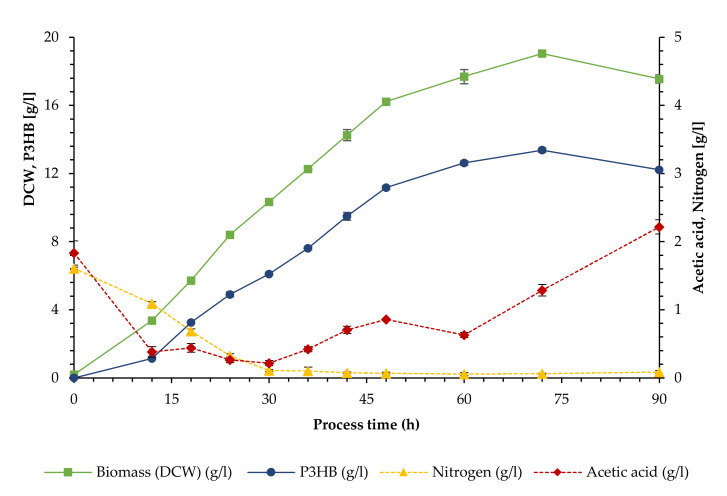
Cultivation of *Bacillus megaterium* uyuni S29 in a cell recycle process (I). Biomass (as DCW) and polymer (P3HB) concentration (left axis) and concentration of acetic acid and nitrogen (as TKN) (right axis). Error bars represent standard deviation from triplicate samples.

This study examined growth and polymer production of *Bacillus megaterium* uyuni S29 from a dilute substrate in a cell recycle setup with a crossflow membrane. Growth and polymer production over the duration of the fermentation process (90 h) are shown in Figure 2. After a lag phase, there is a linear increase in DCW until 48 h. A continued, but less steep ascent can be observed until 72 h, reaching a maximum value of 19.05 g/L, after which there is a decline to 17.55 g/L at the end of the process. Polymer production followed the same profile as biomass, peaking at 72 h with 13.38 g/L (70.21% of DCW) and declining towards the end to 12.25 g/L (69.61% of DCW). The maximal PHB productivity was 0.058 g/Lh, with a corresponding yield of 0.23 g/g reached after 48 h into the process.

Nitrogen was not provided in the feed solution and became a limiting factor after 30 h, when its level dropped below 0.1 g/L. The acetate level in the fermenter stabilized at a level below 1 g/L between 12 and 60 h, with a minimal concentration of 0.22 g/L after 30 h. Thereafter, it slowly increased, reaching its maximal value of 2.22 g/L at the end.

**Figure 3:**
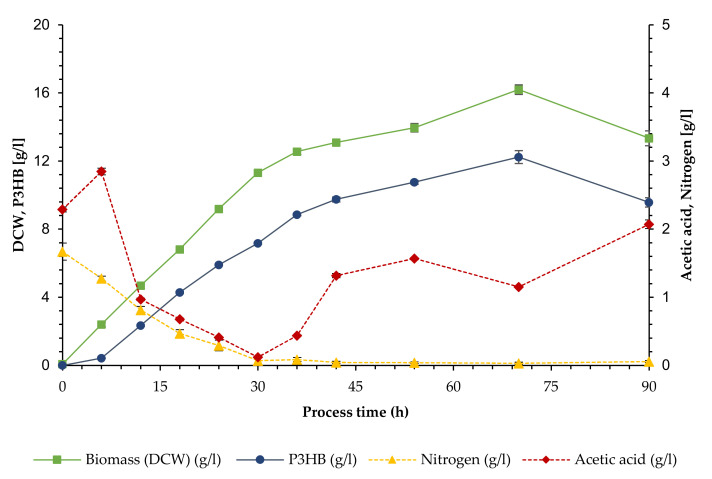
Cultivation of *Bacillus megaterium* uyuni S29 in a cell recycle process (II, replicate). Biomass (as DCW) and polymer (P3HB) concentration (left axis) and concentration of acetic acid and nitrogen (as TKN) (right axis). Error bars represent standard deviation from triplicate samples.

A repetition of the experiment shows similar growth and polymer production (Figure 3). In this case, the linear growth phase was shorter and lasted until 36 h. DCW continued to increase at a smaller rate until 70 h, reaching a maximum value of 16.20 g/L, after which there was a sharp decline for the final value of 13.34 g/L. Polymer production followed the same trend, with low levels already present at the start of the experiment and reflecting course of biomass through the 70 h peak at 12.24 g/L (76% of DCW) all the way to the final concentration of 9.58 g/L (71.78% of DCW). The maximal productivity was 0.062 g/Lh with a corresponding yield of 0.26 g/g, which was again reached when the curve began to flatten (36 h). After 30 h, the nitrogen concentration dropped below 0.07 g/L, indicating limitation. In this experiment, an accumulation of acetic acid was observed after the feed was started, since an additional sampling point after 6 h was added. Acetic acid concentration then decreased steadily to a minimum value of 0.12 g/L at 30 h, and increased thereafter to the final concentration of 2.07 g/L.

The selection of a halophilic strain and the cell retention design open the possibility of non-sterile processes with pure cultures. A high salt concentration combined with a strong and adapted inoculum could keep potential contaminants at bay during the initial phase, while the nutrient limitation would not allow for significant growth of competing organisms in the later stages of the process. This strategy was partially implemented in the present work, where sterilization procedures were applied only for the initial reactor volume and the first 10 L of feed. No contamination could be detected when checked under the microscope.

In the literature, most work on the conversion of volatile fatty acids to PHA relates to the application of mixed microbial consortia. This approach requires a sequential batch approach and, in most cases, results are less favorable compared to pure culture approaches [40,41]. The most successful PHA production from VFAs to date was achieved by Huschner et al. in a regular fed-batch with *Ralstonia eutropha* H16 (different designation for *Cupriavidus necator*) using a concentrated VFA solution resulting in cell densities of 112 g/L with 83% polymer content [18]. The feeding regime was a combination of pH-stat with acid solution and DO-stat with VFA salts. The feed solution contained organic acids in the approximate range of 300–600 g/L and organic salts at about 350 g/L. While ideal for fed-batch processes, it is questionable whether feed concentrations in this range can be obtained from renewable resources in an economically feasible way. More comparable with the present study is the work of Du et al., who achieved 22.7 g/L of DCW with 72.6% polymer in a similar setup with *C. necator*. With respect to the production strain it is notable that the achieved DCW and polymer concentrations in our study are comparable with values for *Bacillus megaterium* on a sugar rich substrate [14].

The large constraint to reaching higher cell densities and productivities in our study was nitrogen content [42]. Due to the way the system is configured, a part of the nitrogen is washed out with the permeate. This design made nitrogen a much more limiting factor compared to the fed-batch version of the same process. Based on the data from nitrogen measurements and feed addition it can be calculated that approximately 0.79 g/L of the initial nitrogen content was consumed by the cells.

In order to improve the productivity [18], it will be necessary to optimize the nitrogen supply to accumulate more biomass [42]. This could be achieved by starting with a higher nitrogen concentration in the medium or by introducing a nitrogen source with the feed solution. However, it must be considered that faster growth will result in higher feed rates and consequently a faster washout of nutrients, which ultimately limits this approach. The addition of nitrogen to the feed should be further investigated, however, this needs to be carefully optimized with nutrient limitations in mind [42].

Due to the inhibitive properties of acetate at elevated concentrations, an overfeed of the system was identified as a potential risk during operation [18,35]. The feeding regime is a calculation-based pH-stat and there is a possibility that small errors in calculation or feed preparation amplify. Therefore, it is critical to align the pH of the feed and pH setting of the process control, as this could easily create a negative or positive feedback loop. However, during operation the system turned out to run stably without any issues in this regard. This could be explained by a partial compensation of changes in broth composition due to the washout via the permeate. Additionally, a low acetate content in the feed prevents the acetate content in the reactor from suddenly increasing in the case of systemic glitches in the process control.

A major disadvantage of VFA as a raw material compared to sugar based substrates is the low productivity [31]. A continuous mode of operation could lead these processes towards higher productivities. MMC processes are currently being rapidly developed in this regard [36,43]. Various pure culture based processes have also been investigated [44,45,46] and the process scheme in the present work would surely benefit in terms of productivity from continuous mode. The most straightforward way to convert this process to continuous mode would the continuous removal of the fermentation broth, creating a chemostat [31]. This should be possible with this strain, since it produces a polymer during the growth stage. There would be no typical PHB accumulation phase, and all of the polymer would have to be produced during exponential growth, requiring a dual limitation strategy [42] and imposing some limit on the desired increase in productivity. Another option would involve the coupling of two cell recycling processes, with a first reactor dedicated for unlimited biomass production and a second reactor with optimal conditions for polymer accumulation. Different volumes in the reactors would determine the residence time/dilution factor to achieve the goals of each step [31].

### 3.2. Membrane Performance

**Figure 4:**
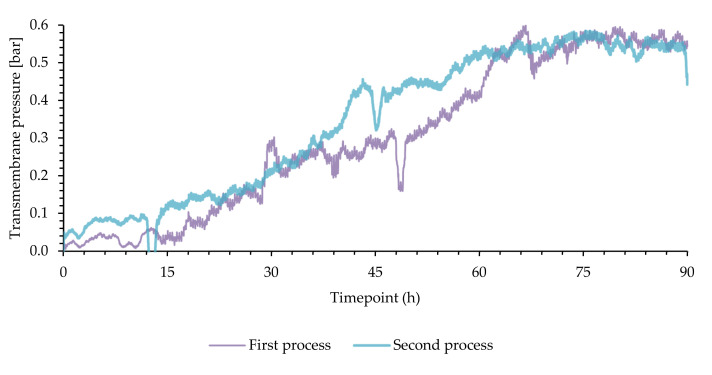
Transmembrane pressure of the two cell recycle processes with *Bacillus megaterium* uyuni S29. The membrane pressure curves are shown as 60 min rolling averages.

The membrane behavior in both experiments is shown in Figure 4. The performance was stable, but there is an increase in transmembrane pressure over time due to fouling. The membrane backflush protocol allowed for uninterrupted operation for the duration of the process. However, if the process reached higher cell densities and required a faster feed rate, an exchange of a membrane module might be necessary. For a practical full-scale application, specialized membrane technology could enable more robust operation, but the module used proved to be sufficiently functional for the lab-scale process.

### 3.3. Polymer Characterization

The polymer was separated from the biomass by digestion of the surrounding biomass using hypochlorite. The obtained molecular weights and results of the DSC (Figure S1, Table S1) and TGA (Figure S2) of the extracted polymer are shown in Table 1 together with values that were obtained from polymers of the same strain in other studies, and commercial products. The results indicate that the grade of the extracted polymer is lower than what might be normally expected from polymers of this strain. This is most likely a consequence of the hypochlorite extraction, which tends to degrade the polymer and reduce the molecular weight. This is also indicated by the PDI, which is very low compared to values previously obtained with the same strain [46]. The thermal properties follow this trend and are considerably poor. Therefore, the possible applications for such a polymer are very limited. As material properties can be influenced by both cultivation and extraction, it is likely that a higher-quality polymer can be obtained with an adapted purification process.

## 4. Conclusions

It could be demonstrated that a cell recycling fed-batch system can be operated in a stable manner to produce P3HB from acetate with *Bacillus megaterium*. While the process indicators were within the anticipated range, there is still room for improvement. The development of a more suitable nitrogen feeding regime is expected to increase biomass concentrations and consequently the productivity of the process. The hypochlorite extraction used for purification had a negative impact on the material properties of the obtained polymer, which underlines the importance of selecting an appropriate extraction process. When applied to waste-derived VFA mixtures, the presented process has the potential to contribute to a sustainable and efficient production of biopolymers.

## Figures and Tables

**Table 1:** Material properties produced by the same strain in different studies in comparison with commercially available products.

Sample Origin	M_w_ [kg/mol]	PDI	T_m_ (°C)	T_g_ (°C)	T_d_ (°C)
This study	56.83	1.29	154.30	−23.27	184.50
This study	59.65	1.43	151.84	−24.02	194.97
*B.megaterium* S29 [47]	350	2.76	178	1.8	238.7
*B.megaterium* S29 [48]	600–125	nd	161	−11	nd
*B.megaterium* S29 [48]	600–125	nd	136.8	−16	nd
Mirel TM F1005 Mirel [49]	71.7	2.1	166.7	nd	283.3
Mirel TM F1006 Mirel [49]	71.4	2.2	165.7	nd	275.5
Enmat Y1000 [49]	77.6	2.6	168.9	nd	272.8

M_w_: weight average molecular weight; PDI: polydispersity index; T_m_: melting temperature; T_g_: glass transition temperature; T_d_: degradation temperature; nd: not determined.

## Data Availability

Essential data are contained within the article. The raw data are available on request from the corresponding author.

## Supplementary Materials

The following supporting information can be downloaded at: https://www.mdpi.com/article/10.3390/bioengineering9030122/s1, Figure S1: Differential scanning calorimetry (DSC) of the extracted polymers; Figure S2: Thermogravimetric analysis (TGA) of the extracted polymers; Table S1: Results differential scanning calorimetry (DSC) of the extracted polymers.
