# Supplementary material for: Cell Retention as a Viable Strategy for PHA Production from Diluted VFAs with Bacillus megaterium
Source: Bioengineering (Basel). 2022 Mar 16;9(3):122. doi: 10.3390/bioengineering9030122 (PMC8945770; doi:10.3390/bioengineering9030122)
Supplement: Supplementary file 1 [file bioengineering-09-00122-s001.zip › bioengineering-1634477-supplementary.pdf]

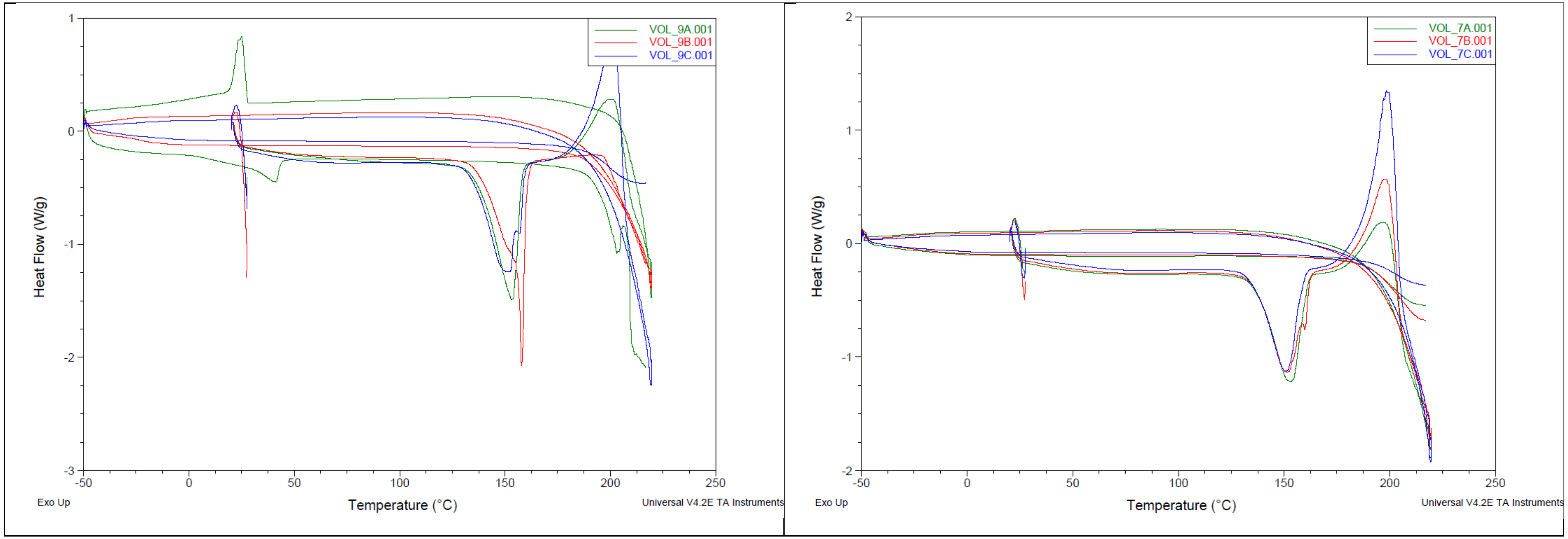


**Figure S1.** Differential scanning calorimetry (DSC) of the extracted polymers (left: process I, right: process II).

**Table S1.** Results differential scanning calorimetry (DSC) of the extracted polymers

| Sample | Sample weight (mg) | $T_m$ (° C) | $\Delta H_m$ (J/g) | $T_g$ (°C) | $T_m$ (°C) | $T_g$ (°C) |
|---|---|---|---|---|---|---|
| Extracted Polymer Process I | 3.2 | 152.98 | 96.54 | -25.34 | 154.30 | -23.27 |
| | 3.3 | 157.80 | 101.10 | -23.80 | | |
| | 3.0 | 152.11 | 98.78 | -20.66 | | |
| Extracted Polymer Process II | 3.3 | 153.03 | 89.93 | -21.77 | 151.84 | -24.02 |
| | 3.1 | 151.60 | 92.68 | -25.18 | | |
| | 3.5 | 150.90 | 83.40 | -25.10 | | |

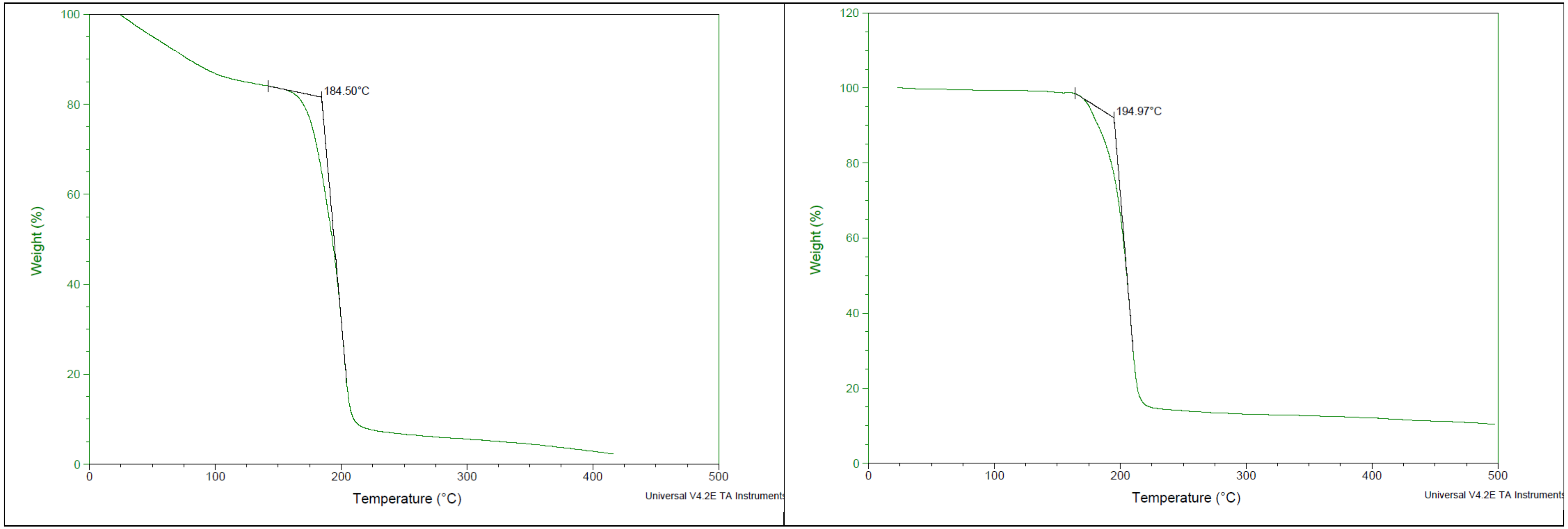


**Figure S2.** Thermogravimetric analysis (TGA) of the extracted polymers (left: process I, right: process II)
